# 2277. Variability in Changes in Physician Outpatient Antibiotic Prescribing from 2019 to 2021 during the COVID-19 Pandemic in Ontario, Canada

**DOI:** 10.1093/ofid/ofad500.1899

**Published:** 2023-11-27

**Authors:** Pranav Tandon, Kevin Brown, Nick Daneman, Bradley J Langford, Valerie Leung, Lindsay Friedman, Kevin Schwartz

**Affiliations:** University of Toronto, Public Health Ontario, Toronto, Ontario, Canada; University of Toronto, Toronto, Ontario, Canada; Sunnybrook Health Sciences Centre, University of Toronto, Toronto, Ontario, Canada; Public Health Ontario, Toronto, Ontario, Canada; Public Health Ontario, Toronto, Ontario, Canada; Public Health Ontario, Toronto, Ontario, Canada; Public Health Ontario Dalla Lana School of Public Health, University of Toronto, Toronto, Ontario, Canada

## Abstract

**Background:**

Outpatient antibiotic prescribing decreased during the COVID-19 pandemic. Understanding how antibiotic prescribing habits changed differentially based on physician and practice characteristics presents an opportunity to inform antibiotic stewardship. Our objective was to evaluate inter-physician variability and predictors of changes in antibiotic prescribing before (2019) and during (2020/2021) the COVID-19 pandemic.

**Methods:**

We conducted a retrospective cohort analysis of physicians in Ontario, Canada prescribing oral antibiotics in the outpatient setting between 1 January 2019 and 31 December 2021 using the IQVIA Xponent dataset. The primary outcome was the change in the number of antibiotic prescriptions between the pre-pandemic and pandemic period. Secondary outcomes were changes in the selection of broad-spectrum agents and long-duration ( >7 days) antibiotic use. We used multivariable linear regression models to evaluate physician- and practice-level predictors of change.

**Results:**

There were 17,288 physicians included in the study with substantial inter-physician variability in changes in antibiotic prescribing (median change of -43.5 antibiotics per physician, IQR -136.5 to -5.0). In the multivariable model, later career stage (adjusted mean difference [aMD] -45.3, 95% confidence interval [CI] -52.9 to -37.8, p< .001), family medicine (aMD -46.0, 95% CI -62.5 to -29.4, p< .001), male patient sex (aMD -52.4, 95% CI -71.1 to -33.7, p< .001), low patient comorbidity (aMD -42.5, 95% CI -50.3 to -34.8, p< .001), and high prescribing to new patients (aMD -216.5, 95% CI -223.5 to -209.5, p< .001) were associated with decreases in antibiotic initiation. Family medicine and high prescribing to new patients were associated with significant decreases in selection of broad-spectrum agents and prolonged antibiotic use.

**Conclusion:**

Antibiotic prescribing changed throughout the COVID-19 pandemic with overall decreases in antibiotic initiation, broad-spectrum agents, and prolonged antibiotic courses with inter-physician variability. These findings present opportunities for targeted community antibiotic stewardship interventions.

**Disclosures:**

**All Authors**: No reported disclosures

